# Cystic Fluid Total Proteins, Low-Density Lipoprotein Cholesterol, Lipid Metabolites, and Lymphocytes: Worrisome Biomarkers for Intraductal Papillary Mucinous Neoplasms

**DOI:** 10.3390/cancers17040643

**Published:** 2025-02-14

**Authors:** Fahimeh Jafarnezhad-Ansariha, Nicole Contran, Chiara Cristofori, Manuela Simonato, Veronica Davanzo, Stefania Moz, Paola Galozzi, Paola Fogar, Evelyn Nordi, Andrea Padoan, Ada Aita, Matteo Fassan, Alberto Fantin, Anna Sartori, Cosimo Sperti, Alessio Correani, Virgilio Carnielli, Paola Cogo, Daniela Basso

**Affiliations:** 1 Department of Surgery, Oncology and Gastroenterology-DISCOG, University of Padua, 35128 Padua, Italy; fahimeh.jafarnezhadansariha@studenti.unipd.it (F.J.-A.); cosimo.sperti@unipd.it (C.S.); 2 Laboratory Medicine, University-Hospital of Padua, 35128 Padua, Italy; nicole.contran@studenti.unipd.it (N.C.); veronica.davanzo@studenti.unipd.it (V.D.); stefania.moz@aopd.veneto.it (S.M.); paola.fogar@aopd.veneto.it (P.F.); evelyn.nordi@studenti.unipd.it (E.N.); andrea.padoan@unipd.it (A.P.); ada.aita@unipd.it (A.A.); daniela.basso@unipd.it (D.B.); 3 Department of Gastroenterology, Veneto Institute of Oncology IOV-IRCCS, 35128 Padua, Italy; chiara.cristofori@iov.veneto.it (C.C.); alberto.fantin@iov.veneto.it (A.F.); 4 Department of Medicine—DIMED, University of Padua, 35128 Padua, Italy; lab2nut@gmail.com (M.S.); matteo.fassan@unipd.it (M.F.); 5 Pediatric Research Institute “Citta’ della Speranza”, Critical Care Biology and PCare Laboratories, 35127 Padua, Italy; anna.sartori.10@phd.unipd.it; 6 Department of Odontostomatologic and Specialized Clinical Sciences, Polytechnic University of Marche, 60131 Ancona, Italy; alessio.correani@studenti.unipd.it (A.C.); v.carnielli@gmail.com (V.C.); 7 Division of Neonatology, Mother and Child Department, G. Salesi University Hospital, 60123 Ancona, Italy; 8 Department of Medicine, Division of Pediatrics, S. Maria della Misericordia University Hospital, University of Udine, 33100 Udine, Italy; paola.cogo@uniud.it

**Keywords:** amylase, carcinoembryonic antigen, hematopoietic stem/progenitor cells, metabolomics, mucinous cystic neoplasms, pancreatic cystic neoplasms

## Abstract

**Objectives:** Pancreatic cystic neoplasms (PCNs), particularly intraductal papillary mucinous neoplasms (IPMNs), present a challenge for their potential malignancy. Despite promising biomarkers like CEA, amylase, and glucose, our study investigates whether metabolic indices in blood and cystic fluids (CFs), in addition to lymphocyte subsets and hematopoietic stem/progenitor cells (HSPCs), can effectively differentiate between high- and low-risk PCNs. **Materials and Methods:** A total of 26 patients (11 males, mean age 69.5 ± 9 years) undergoing Endoscopic Ultrasound-guided Fine Needle Aspiration were consecutively enrolled. Analyses included blood, serum, and CF, assessing glucose, CEA, cholesterol (total, HDL, and LDL), and total proteins. Flow cytometry examined immunophenotyping in peripheral blood and cystic fluids. Mass spectrometry was used for the metabolomic analysis of CF. Sensitivity, specificity, and ROC analyses evaluated discriminatory power. **Results:** A total of 25 out of 26 patients had IPMN. Patients were categorized as low or high risk based on multidisciplinary evaluation of clinical, radiological, and endoscopic data. High-risk patients showed lower CF total proteins and LDL cholesterol (*p* = 0.005 and *p* = 0.031), with a marked reduction in CF lymphocytes (*p* = 0.005). HSCPs were absent in CF. In blood, high-risk patients showed increased non-MHC-restricted cytotoxic T cells (*p* = 0.019). The metabolomic analysis revealed significantly reduced middle and long-chain acyl carnitines (AcCa) and tryptophan metabolites in high-risk patients. ROC curves indicated comparable discriminant abilities for CF lymphocytes (AUC 0.868), CF total proteins (AUC 0.859), and CF LDL cholesterol (AUC 0.795). The highest performance was achieved by the AcCa 14:2 and 16:0 (AUC: 0.9221 and 0.8857, respectively). **Conclusions:** CF levels of glucose, CEA, LDL cholesterol, and total proteins together with lymphocyte counts are easy translational biomarkers that may support risk stratification of PCNs in IPMN patients and might be endorsed by metabolomic analysis. Further studies are required for potential clinical integration.

## Simple Summary

Pancreatic cystic neoplasms, such as intraductal papillary mucinous neoplasms (IPMNs), often pose diagnostic challenges due to their varying malignancy risks. Existing biomarkers, while helpful, lack precision, necessitating better tools for risk stratification. This study explores the potential of combining metabolic indices, lymphocyte profiles, and advanced metabolomic analyses to distinguish high-risk IPMNs from low-risk cases. By analyzing cystic fluid and blood samples, key indicators such as cystic fluid LDL cholesterol, total proteins, and lymphocytes were identified as promising markers for malignancy risk. These findings could improve clinical decision making, paving the way for more accurate diagnostics and earlier interventions in pancreatic cancer.

## 1. Introduction

Pancreatic cystic neoplasms (PCNs), frequently identified incidentally, necessitate clinical decision making due to their wide spectrum of lesions, encompassing benign, pre-neoplastic, and malignant entities [1,2,3,4]. The incidental discovery rate of PCNs is approximately 2% in patients undergoing CT, rising to 13–45% in those subjected to MRI/MRCP, likely attributable to the latter’s superior resolution [2]. When PCNs are detected, differentiating between benign and potentially malignant lesions is required to guide the most appropriate management, which includes follow-up strategies without surgery indication and instances where surgery is either absolutely or relatively indicated for high-malignant-risk cases [1,2,4]. While certain radiological features, such as dilation of the main pancreatic duct and enhancing mural nodules, are predictive of malignancy, the overall accuracy of radiology in distinguishing specific PCN types remains limited [2,4]. To enhance the prediction rate of PCN malignancy, especially in cases with concerning clinical or radiologic features, endoscopic ultrasonography coupled with fine-needle aspiration (EUS-FNA) for cystic fluid analysis, including CEA and amylase/lipase measurements and cytology, is recommended [2,5,6,7]. However, the overall diagnostic accuracy of EUS-FNA with cystic fluid analyses does not exceed 70% in distinguishing benign from malignant PCNs [8]. Cytology, while highly specific, exhibits limited sensitivity. Typically, mucinous PCNs are likely to be malignant and serous PCNs benign [9]. CEA, amylase, and lipase measurements in cystic fluid might help in distinguishing mucinous from non-mucinous lesions, with higher CEA and lower amylase/lipase levels observed in mucinous types. The recommended cystic fluid CEA threshold to discriminate mucinous pancreatic cystic neoplasms (MCNs) from non-mucinous PCNs is 192 ng/mL, with a sensitivity of 52–78% and a specificity of 63–91% [2,10]. More recently, Sharma et al. proposed a CEA threshold of 45 ng/mL, reporting a sensitivity and specificity of 89% and 97%, respectively [11]. Molecular markers, such as somatic mutations of KRAS and GNAS, have also been studied, the former being commonly found in both intraductal papillary mucinous neoplasms (IPMNs) and MCNs, the latter being strictly correlated with MCNs. Their routine analyses are not yet recommended due to the need for highly equipped laboratories and trained personnel [2,8,12,13].

The identification of new, cost-effective, effortless biomarkers able to distinguish mucinous from non-mucinous PCNs is a current challenge. The two most prevalent PCNs, IPMNs and MCNs, pose a risk of malignant transformation, particularly when growing rapidly and exceeding 40 mm in size [2]. The progression of IPMNs to pancreatic cancer involves mild-moderate-severe dysplasia with accumulating genetic and epigenetic alterations, including EGFR over-expression and mainly mutations in KRAS, p53, and SMAD4 [12,14]. The oncogenic switch, marked by the accumulation of genetic and epigenetic alterations, harbors additional hallmarks, including altered cell metabolism and a suppressed anti-cancer immune response [15]. The Warburg effect or aerobic glycolysis is a hallmark of cancer cell metabolism [16,17,18]. Altered glucose metabolism is a frequent finding in pancreatic cancer, with early-onset diabetes considered a clinical manifestation and a sign of malignancy in the case of PCNs [19,20]. Conversely, reduced glucose levels in the cystic fluid are suggested to correlate with a higher risk of malignancy [21]. In order to identify new potential biomarkers of malignancy related to the metabolic reprogramming of cancer cells, the metabolomic approach has also been used. This approach highlighted that in cystic fluid, besides glucose, amino acids and lipid metabolites such as kynurenine, 5-oxoproline, free fatty acids, and ceramides are potential predictors of malignancy [22].

Cancer cells might evade immunosurveillance by downregulating the expression of antigenic molecules, causing an imbalance in lymphocyte subsets that favors the expansion of tolerogenic and immunosuppressive cells, thus creating a conducive environment for cancer cell growth and invasion [23,24]. Moreover, in vitro studies indicate pancreatic cancer cells inhibit lymphocyte proliferation, and in vivo, a reduced number of circulating lymphocytes predicts an adverse outcome, both independently and when included in derived indexes, such as platelet/lymphocyte ratio (PLR) and neutrophil/lymphocyte ratio (NLR) [25,26]. Hematopoietic stem/progenitor cells potentially play a role in the complex cellular interplay connecting cancer, diabetes, and immunosuppression given their sensitivity to glucose, potential impact on NLR, and documented increased trafficking in pancreatic cancer [27,28].

The aim of this study was to investigate whether blood and cystic fluid levels of the most commonly employed metabolic indices of energy metabolism, lymphocyte subsets, and hematopoietic stem/progenitor cells could aid in distinguishing between high-risk mucinous and low risk non-mucinous PCNs. To this aim, we also investigated the hydrophilic metabolic profile of cystic fluid samples.

## 2. Materials and Methods

### 2.1. Patients

A total of 26 patients, comprising 11 males and 15 females with a mean age ± SD of 69.5 ± 9.6 year (ranging from 48–84 years), were consecutively recruited from the Department of Gastroenterology, Veneto Institute of Oncology IOV-IRCCS, during the period from February 2022 to December 2023. The study was conducted according to the guidelines of the Declaration of Helsinki and approved by the Institutional Review Board of the University-Hospital of Padua (AOP0718), and written informed consent was obtained from all participants. All patients underwent EUS-FNA for pancreatic cystic neoplasms. Among the 26 patients, 16 presented with a singular cyst, distributed as follows: 8 in the head, 1 in the body, 1 in the tail, 2 at the head-uncinate process, 1 at the body-tail, 2 at the uncinate process, 1 at the neck. The remaining 10 patients exhibited multiple cysts, distributed as follows: 2 at the head, 2 at the head-uncinate process, 2 at the body tail, 1 at the tail and head, and 1 at the body and head. Cystic fluid was successfully obtained whenever possible from 24 out of 26 patients, prioritizing the largest cyst in cases of multiple cysts. The mean ± SD diameter of cysts was 3.3 ± 0.9 cm, ranging from 2 to 5 cm. Subsequently, the cystic fluid underwent the string sign test, followed by cytology, carcinoembryonic antigen (CEA), and amylase measurements. Blood samples were obtained before endoscopy and analyzed for complete blood count, flow cytometry, biochemistry, and serum carbohydrate antigen 19-9 (CA19-9). All biological samples were managed within 1 h of sampling. Flow cytometry and biochemistry analyses of blood and cystic fluids were conducted on the collection day, and the remaining samples were aliquoted and promptly frozen at −80 °C for further investigations.

### 2.2. Clinical Laboratory Assessments

Routine hematological parameters included red blood cell count (RBC); hemoglobin concentration (Hb); hematocrit (HCT); mean corpuscular volume (MCV); mean corpuscular hemoglobin (MCH); mean corpuscular hemoglobin concentration (MCHC); platelet count; and counts for neutrophils, lymphocytes, and monocytes (XN-Series, Sysmex, Kobe, Japan). Plasma biochemical parameters were analyzed using the Cobas e702 analyzer (Roche Diagnostics S.p.A., Monza, Italy), encompassing aspartate aminotransferase (AST); alanine aminotransferase (ALT); alkaline phosphatase (ALP); gamma-glutamyl transferase (GGT); and total, conjugated, and non-conjugated bilirubin. The same analyzer was used to measure cyst fluid amylase, plasma and cystic fluid glucose, urea, total cholesterol, HDL (high-density lipoprotein) cholesterol, LDL (low-density lipoprotein) cholesterol, triglycerides, and total proteins. Additionally, CEA and CA19-9 levels were determined in both serum and cystic fluid using LIAISON^®^ CEA and CA19-9™ kits (DiaSorin S.p.A., Saluggia, Italy) by chemiluminescent immunoassays.

### 2.3. Lymphocyte Subsets and Hematopoietic Stem/Progenitor Cells

Phenotypic analyses of mature T cells, T helper inducer cells, Cytotoxic T cells, B cells, NK cells, and non-MHC-restricted cytotoxic cells in peripheral blood were performed using the Aquios CL flow cytometer and the antibody mixtures AQUIOS Tetra-1 and AQUIOS Tetra-2+ (Beckman Coulter, Brea, CA, USA). Hematopoietic stem/progenitor cells (HSPCs) were analyzed in blood and cystic fluid with the DxFlex flow cytometer (Beckman Coulter, CA, USA) using the following antibodies: PE (Phycoerythrin) labeled anti-CD34 antibody (mouse monoclonal IgG1, clone 581), ECD (Phycoerythrin-Texas red tandem conjugate) labeled anti-CD45 antibody (mouse monoclonal IgG1, clone J33), and APC (allophycocyanin) labeled anti-CD133 antibody (mouse monoclonal IgG1, clone W6B3C1) (Beckman Coulter, CA, USA). HSPC flow cytometry was performed after obtaining the number of total leukocytes (WBC/mL) in blood and cystic fluid (XN-Series, Sysmex, Kobe, Japan). Cell analysis was made following the lyse-and-wash procedure. For each sample, a minimum of 1 × 10^6^ cells was recorded, and data were analyzed through the software Kaluza Analysis 2.1 (Beckman Coulter, CA, USA).

The absolute number of HSPCs × 109/L was calculated using the following Formula (1):

(1) $$\frac{\left(number\;of\;events\;CD34+CD133+CD45+weak)\times (number\;of\;WBC\times 10^{9}/L\right)}{\left(number\;of\;total\;CD45+events\right)}$$

The presence and distribution of leukocytes in the cystic fluid was also performed within the setting of HSPC analysis. Side (SSC) and forward scatter (FSC) together with CD45 expression intensity were used to define lymphocytes, monocytes, and neutrophils. The percentage of lymphocytes was calculated based on the ratio between the number of events in the lymphocyte gate over the number of events in the leukocyte gate.

### 2.4. Metabolomic Analysis

A total of 18 (*n* = 7 mucinous high risk and *n* = 11 non-mucinous low risk) cystic fluid samples were analyzed by mass spectrometry following the protocol indicated in the Supplementary Materials.

Metabolite extraction: First, a 40 µL aliquot of cystic fluid sample was treated with 120 µL of ice-cold solvents (50:50 ACN:H_2_O or 50:50 MeOH:H_2_O). The mixture was vortexed for 2 min and then centrifuged at 13,000× *g* for 20 min at 4 °C to precipitate the proteins. The supernatant layer was transferred into autosampler vials for metabolite analysis. Quality control (QC) samples were prepared by mixing equal volumes of all the cystic fluid samples. These pooled QC samples were prepared as described for real samples. A procedural blank, used to monitor contamination acquired during all stages of sample preparation, and a pool of the 7 high- and 11 low-risk samples, were also prepared.

The column compartment and autosampler were maintained at temperatures of 35 °C and 15 °C, respectively. Injection volumes were set to 3 µL for the positive ionization mode and 5 µL for the negative ionization mode. To avoid potential biases related to sample positioning within the sequence, samples were injected in duplicate in a randomized order. Liquid chromatography separation: HILIC and reverse phase (RP) separations were performed on a Dionex UltiMate 3000 RS system coupled to a Q Exactive Classic mass spectrometer equipped with a heated-ESI-II (HESI-II) ion source operating in positive and negative polarity (Thermo Fisher Scientific, Waltham, MA, USA). An Accucore 150 Amide column (2.1 mm × 100 mm, 2.6 μm, Thermo Fisher Scientific) was used for HILIC separation. Mobile phase A was ACN:H_2_O (95:5, *v*/*v*) and mobile phase B was ACN:H_2_O (50:50, *v*/*v*) both modified with 10 mM ammonium formate and 0.1% of formic acid. The column was eluted with a linear gradient from 1–15% B over 15 min, a linear gradient to 95% B over 10 min, isocratic conditions at 95% B for 2 min, a linear gradient to 1% of B over 0.5 min, and isocratic conditions at 1% B for 5.5 min at a flow rate of 450 µL/min. A Hypersil Gold C-18 column (100 × 2.1 mm, 3.0 μm, Thermo Fisher Scientific) was utilized for RP separation. The elution was carried out at a flow rate of 300 µL/min, starting with 80% mobile phase A (0.02% formic acid in water) for 0.1 min followed by a linear gradient to 100% mobile phase B (0.02% formic acid in methanol) over 20 min. This condition was held constant for 5 min, then returned to the initial condition within 0.5 min and maintained for an additional 4 min.

Data analysis: Raw data files were processed by the Compound Discoverer™ 3.3.2 software for initial data processing, including peak detection, peak alignment, and peak integration. Raw files were aligned with adaptive curve setting with a 5 ppm mass tolerance and 0.4 min retention time shift.

Unknown compounds were identified using a mass tolerance of 5 ppm, a signal-to-noise ratio of 3, and a relative intensity tolerance of 30% for isotope searches. These compounds were then grouped based on a mass tolerance of 5 ppm and a retention time tolerance of 0.2 min. Background subtraction and noise removal were performed during preprocessing using a procedural blank sample. Peaks showing less than a 3-fold increase compared to the blank sample, detected in fewer than 50% of QCs, or with a relative standard deviation (%RSD) exceeding 30% in QCs were excluded. Peak areas across all samples were normalized to the total area of their corresponding samples to account for intensity variations caused by instrument instability. Metabolites detected in the processed raw mass spectral data were cross-referenced against the ChemSpider™ chemical structure database (3 ppm mass tolerance) and the mzCloud and mzVault spectral libraries (precursor and fragment mass tolerance, 10 ppm). Five data sources were selected from the ChemSpider database: Human Metabolome Database (HMDB), Kyoto Encyclopedia of Genes and Genomes (KEGG), LipidMAPS, Biocyc, and DrugBank. Lipid identity confirmation was conducted using Lipid Search™ 5.

The chemical analysis working group categorized metabolite identification into four levels, as commonly described in the scientific literature [29]: identified metabolites (level 1), putatively annotated compounds (level 2), putatively characterized compound classes (level 3), and unknown compounds (level 4). Our discriminating metabolites were all identified by both the molecular formula and the fragmentation data (Level 2). Only significant metabolites were selected for further pathway analysis using the free web-based software MetaboAnalyst (Version 6.0).

### 2.5. Statistical Analysis

The Shapiro–Wilk test was used to ascertain whether data had a normal distribution or not. Student’s *t*-test and Kruskal–Wallis non-parametric testing were conducted. The correlation coefficients were analyzed using Pearson’s and Spearman’s tests for parametric and non-parametric data, respectively. Receiver operating characteristic (ROC) curves were used to evaluate tests performance in distinguishing patient categories. All statistical analyses were performed by the Stata software v13.1 (Statacorp, College Station, TX, USA) and GraphPad Prism v10 (GraphPad, San Diego, CA, USA). A two-tailed *p*-value of <0.05 was considered statistically significant.

## 3. Results

### 3.1. Patient Risk Stratification

A total of 26 patients were included in the study: 24 IPMN, 1 mixed typed IPMN, and 1 serous cystadenoma.

Two patients presented with dysplasia on cytology; however, no other high-risk features indicative of HGD/IC were identified, such as jaundice, an enhancing mural nodule ≥ 5 mm or solid component, or a main pancreatic duct ≥ 10 mm. In accordance with the Kyoto and Fukuoka guidelines [30,31], worrisome features were assessed as outlined in Supplementary Table S1. A multidisciplinary team involving expert gastroenterologists, surgeons, and pathologists collaborated on evaluating all clinical, radiological, and endoscopic information available to classify patients as belonging to the high- or low-risk group. Table 1 shows the demographic, clinical, and circulating laboratory data of the studied patients after subdividing them into two categories with high-risk mucinous or low-risk non-mucinous PCNs. Overall, no significant difference for gender between high- and low-risk patients was found, but a significant difference for age and serum CA 19-9 was detected in older high-risk patients with higher CA 19-9 levels than low-risk patients.

### 3.2. Biochemical Indices in Cystic Fluid

**Figure 1:**
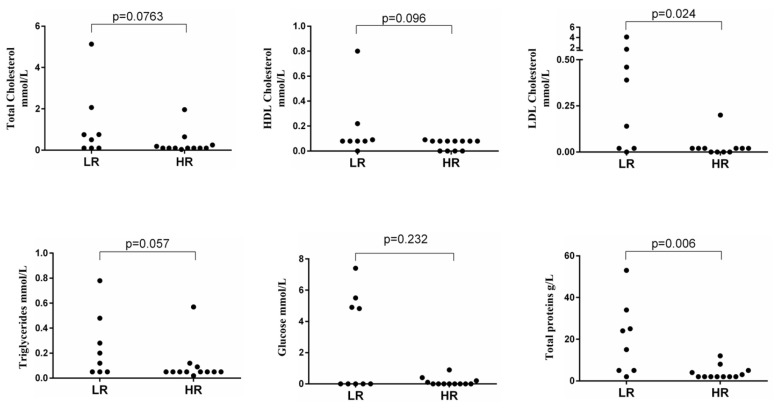
Cystic fluid biochemical parameters in high- and low-risk IPMN patients. *p* < 0.05 was considered significant. LR, low-risk; HR, high-risk.

The biochemical parameters amylase; glucose; total, LDL, and HDL cholesterols; triglycerides; and total proteins were measured in both cystic fluid and plasma. Figure 1 shows cystic fluid findings in low- and high-risk patients as a result of the statistical analyses (Kruskal–Wallis test).

Significantly lower values of total proteins and LDL cholesterol were observed in high-risk with respect to low-risk patients. While the other parameters tended to be lower in high-risk patients, this did not reach statistical significance. Cystic fluid amylase did not differ between high- and low-risk PCNs (Kruskal–Wallis chi square: 0.494, *p* = 0.482). The above studied cystic fluid biochemical markers were all correlated with each other and with cystic fluid lymphocytes (Supplementary Table S2). All biomarkers, but not total proteins, were also inversely correlated with cystic fluid amylase. Age was inversely correlated with total proteins (r = −0.451, *p* = 0.046) and LDL cholesterol (r = −0.462, *p* = 0.047). The size of cystic lesions was correlated with cystic fluid amylase (spearman’s r = −0.686, *p* = 0.005) but not with the other studied biochemical parameters. Plasma levels of the biochemical parameters were not correlated with the cystic fluid corresponding data.

### 3.3. Immune Cells in Cystic Fluid and Blood

HSPCs were never detected in cystic fluid. In contrast, lymphocytes were identified with a significant difference between high- and low-risk patients (chi square = 7.690, *p* = 0.0056), being almost absent in the cystic fluid of high-risk patients. Cystic fluid lymphocytes were inversely correlated with age (r = −0.496, *p* = 0.026) but not with cyst size (r = −0.103, *p* = 0.666). In blood, we evaluated both the common hematological parameters, lymphocyte subsets, and HSPCs (Table 2). Non-MHC restricted cytotoxic T cells were significantly higher in high- than in low-risk patients, and HSPCs tended to be lower in high-risk patients, although the difference was not significant. In 69% (18/26) of patients, independently from risk, hemoglobin was lower than the reference range. None of the blood cells were correlated with age.

### 3.4. Cystic Fluid Biomarkers’ Discriminant Ability

The overall discriminant ability of each cystic fluid biomarker in distinguishing low- from high-risk PCNs was evaluated by ROC curves, the results being reported in Table 3.

Lymphocytes, total proteins, and LDL cholesterol had the highest AUCs, which were comparable to that of CEA. Based on the best combination between sensitivity and specificity with the best percentage of correctly classified patients, a cut-off was identified for any cystic fluid biochemical marker. Using these cut-offs, sensitivity, specificity, positive and negative likelihood ratios, and overall accuracy were calculated for those biomarkers with an AUC above 0.75, namely glucose, LDL cholesterol, total proteins, and lymphocytes. The results are reported in Table 4. For CEA, we used the recommended 192 μg/L cut-off value, and the 52 μg/L cut-off was identified as the best value for the ROC analysis.

### 3.5. LC-MS Results

HILIC metabolites: A total of 6268 and 5105 compounds with unique molecular weight and retention times were annotated from the positive and the negative modes, respectively. After data cleaning to remove unreproducible features (>30% RSD of QC) and background subtraction, 1701 (ESI+) and 1414 (ESI-) compounds remained. Of these 520 and 422 compounds had a *p* value < 0.05 and a group area fold change (expressed as log2) <−1 or >1 (Volcano Plot, Figure S1, upper panels).

Reverse phase (RP) C18 metabolites: A total of 13,637 and 5078 compounds with unique molecular weight and retention times were annotated from the positive and the negative modes, respectively. After data cleaning, 1150 (ESI+) and 871 (ESI-) compounds remained. Of these, 247 and 186 compounds had a *p* value < 0.05 and a group area fold change (expressed as log2) <−1 or >1 (Volcano Plot, Figure S1 lower panels).

Identification of differential metabolites: Among the 942 (HILIC) and 433 (RP C18) discriminant compounds, our analysis led to the identification of 92 metabolites in human cystic fluid samples. The complete list of discriminant metabolites is tabulated in a downloadable file (Supplementary Table S3), while the most representative are reported in Table 5.

Among these metabolites, it was notable that a large proportion was represented by middle and long-chain acyl carnitines, almost all being significantly lower in cystic fluid derived from high-risk patients. Furthermore, we observed altered metabolites linked to the tryptophan metabolism (Kynurenine, methyl indole 3 acetate, and indole 3 lactic acid). After selecting the most significantly different metabolites between mucinous high-risk and non-mucinous low-risk PCNs, for each of them, a ROC curve analysis was performed. Those metabolites with an area under the ROC curve (AUC) higher than 0.88 (*n* = 9), i.e., an AUC higher than that of CEA (0.8786, see Table 3), are reported in Table 6.

By combining the two metabolites with the highest AUC, AcCa (14:2) and 9-HpODE, we were able to distinguish mucinous high-risk from non-mucinous low-risk cysts with and AUC of 0.9351 ± 0.0668. The nine identified metabolites were correlated with the cystic fluid biochemical indices triglycerides, LDL cholesterol, total proteins, and CEA, the results being shown in Supplementary Table S4. ROC curves were then performed by combining the selected metabolites with triglycerides or bilirubin as predictor variables, the results being reported in Supplementary Table S5. For a better understanding of metabolic dysregulation in the cystic fluid of low- and high-risk patients, two types of pathway analysis were performed. Metabolite set enrichment analysis was conducted using RaMP-DB (Figure S2a) and metabolic pathway analysis using the KEGG database, which also calculates the impact of each pathway using a topology analysis in addition to the classic enrichment analysis (Figure S2b).

## 4. Discussion

There is a rising interest in identifying innovative biomarkers for diagnosing malignancy when a PCN occurs as an incidental finding or when it is under active surveillance. As age advances, the disease tends to progress [32], and this concept is in line with our finding that high-risk patients were significantly older than those with low risk. Age difference might be a possible bias, although the mean difference was about 10 years in a cohort of patients that for the vast majority were older than 60 years, a range that has minimal effects on biochemical parameters. Although recommended cystic fluid biomarkers, such as CEA, amylase, and glucose, contribute to diagnosing mucinous neoplasms with a higher risk of malignancy, their overall accuracy does not exceed 80% [3,5,21,33,34]. It should be noted that, in agreement with previous studies, cystic fluid CEA better discriminates high- from low-risk PCNs, lowering the recommended 192 μg/L cut-off to 52 μg/L, which is very close to the value of 45 ng/mL reported by Sharma et al. [11]. Additionally, non-invasive biomarkers measurable in blood or other easily obtainable body fluids could enhance diagnostic opportunities and improve patient compliance. The transition from pre-malignant to malignant lesions involves accumulated genetic and epigenetic alterations in cancer cells, modified energy metabolism, primarily aerobic glycolysis, and an altered anti-tumor immune response, where immunosuppression plays a dominant role [35,36]. These considerations prompted an investigation into cystic fluid lipids and proteins, glucose, and immune cell subtypes in both cystic fluid and blood to identify potential biomarkers of malignancy. Moreover, an extended metabolomic analysis was also performed in cystic fluid.

In our series of IPMN patients, plasma glucose levels did not distinguish high-risk from low-risk individuals, and the prevalence of diabetes in the two cohorts was similar and in line with previous data in the literature [20]. However, cystic fluid glucose levels tended to be lower in high-risk patients, although the difference did not reach statistical significance. The limited discriminative capacity of glucose in our series, confirmed also by ROC curve analyses, is apparently in contrast with previous findings in the literature [21,22,37,38,39,40]. In this case, the reduced levels of glucose further support the concept that cancer cells employ several strategies, including glycolysis regulation and enhanced glucose consumption, to meet energetic and proliferative demands [18,41].

It is believed that cancer cells’ metabolic reprogramming involves not only glucose but also lipid and amino acid metabolism, cancer cells being prone to uptake and use readily available sources rather than spend energy to synthesize de novo the necessary fatty acids and amino acids [42,43,44]. In line with this, high-risk IPMN exhibited lower levels of the cystic fluid lipids, particularly LDL cholesterol, with a sensitivity of 100% at 50% specificity. Mechanistically, this finding might reflect the relevant role of LDL receptor overexpression described in different cancer types, including pancreatic cancer [43,45]. It has been suggested that triggering LDL receptors results in an intratumoral cholesterol imbalance and improves the efficiency of chemotherapies. Notably, the discriminant potential of cystic fluid lipids, especially LDL, highlights their significance as vital structural components of cancer cell membranes and their potential reprogramming during tumor growth [46,47].

The association between an altered lipid profile and PCN risk was also evidenced by the metabolomic analysis and pathway analysis of cystic fluids. The metabolites which differentiate high- from low-risk cystic fluid samples were found to be related to key KEGG categories such as ‘amino acid metabolism’ and ‘glycerophospholipid metabolism’. Lysophosphatidylcholines (LPC 15:1, LPC 16:0, and LPC 17:1) were significantly lower in high-risk than low-risk PCNs. Similar findings for LPC 16:0 were reported by Shi et al. [48], who found a significant reduction of this metabolite in malignant with respect of non-malignant mucinous cysts. Reduced levels of lysophosphatidylcholines have been reported to be associated with several diseases, including pancreatic cancer, diabetes, and obesity, and with worse disease outcomes [49,50,51]. The reduction of LPCs in cystic fluid might contribute to enhancing cancer risk because of their anti-oxidative, pro-apoptotic, and anti-migration effects on cancer cells [49]. In this study, we also found reduced levels of medium-chain (C6-C12) and long-chain (C14-C18) Acylcarnitines (AcCa) in high-risk compared to low-risk PCNs, further supporting that metabolic reprogramming of cancer cells targets lipids as well as glucose metabolism. AcCa is involved in the transport of fatty acids into the mitochondria; reduced levels of medium and long AcCA have been described in several solid tumors, and the role of hypoxia and acidosis as drivers has been hypothesized [52].

In addition to glucose and lipids, the anabolic metabolism of cancer cells results in increasing amino acid uptake through the overexpression of cell membrane transporters but also in increasing protein capture through micropinocytosis [35]. Our findings show a marked significant reduction in the cystic fluid tryptophan metabolites kinurenin, methyl-indole-3-acetate, and indole-3-lactic acid, and total protein levels in high-risk compared to low-risk patients support the above notion and could be considered the expression of a strategy of pre-malignant cells to provide their enhanced protein synthesis needs for maintaining homeostatic processes, rapid growth, and ultimately tumorigenesis [35]. Notably, total protein measurement in cystic fluid demonstrated higher accuracy than CEA, suggesting its promise as a biomarker for assessing the risk of malignancy. Total proteins and LDL cholesterol measurements in cystic fluid might be easily translated in clinical practice to enhance the diagnostic potential of CEA and glucose measurements; on the other hand, metabolites, including long-chain AcCa, appear very promising as new potential diagnostic tools for their very high accuracy in distinguishing low- from high-risk PCNs.

Although efforts have gone into studying various aspects of IPMN, the circulating and cystic fluid immune status of IPMN patients remains largely unexplored. Analyses of main circulating immune cell subsets were conducted focusing on whether alterations occur in high- and low-risk patients. The main immune cell subtypes, namely neutrophils, lymphocytes, monocytes, B cells, NK cells, mature T cells, and T helper inducer cells, did not vary between high- and low-risk IPMNs, nor they fall outside their respective reference intervals, unlike hemoglobin, which was lower than reference values in both high- (64% of cases) and low-risk (75%) patients. Mild anemia, therefore, appears in IPMNs as it occurs in a diverse range of cancers, especially PDAC, and it should be cautiously evaluated [53].

Very little is known about the role of circulating non-MHC-restricted cytotoxic cells in IPMNs. These cells were found to be significantly increased in high-risk compared to low-risk patients. Interestingly, Daley et al. [54] demonstrated that non-MHC-restricted γδT cells infiltrate both the established PDAC microenvironment and pre-invasive murine tumors, promoting oncogenesis and acting as important regulators of effector T-cell activation, favoring immunosuppression. Thus, assessing this circulating immune component could be considered as a potential predictive marker for differentiating IPMNs. However, additional comprehensive studies are required in larger populations.

The role of HSPCs, as multipotent cells with self-renewal abilities, immunomodulatory potential, and differentiation into various cell lineages, in the initiation and progression of pancreatic malignancy, specifically IPMNs, remains controversial. Recent studies have shown that HSPCs can stimulate angiogenesis through high-level secretion of VEFG; upregulation of Jagged-1, a major ligand of Notch signaling in cancer cells; and regulation of the epithelial–mesenchymal transition (EMT), promoting a metastatic phenotype and cancer progression [55,56]. Moreover, HSPCs contribute to the regeneration of insulin-producing β-cells, leading to islet survival in diabetes studies [57,58]. In our research, HSPCs were not detected in cystic fluids, and in blood they did not vary between high- and low-risk IPMNs. However, the assessment of cystic fluid lymphocytes provided a relevant clue for distinguishing high- from low-risk IPMN patients, with almost undetectable levels in the former group. This finding is consistent with the known effect of PDAC on lymphocytes, where the circulating number progressively falls as PDAC worsens, representing a negative prognostic index [25]. The decrease in cystic fluid of high-risk pre-malignant lesions might be the expression of the complex interaction between incipient cancer and stromal cells and, as discussed for lipids and glucose, could also be a novel potential marker of malignancy.

The main strength of our study is the identification of new potential biomarkers of PCN risk of malignancy that are easy to perform in clinical laboratories and of a metabolomic profile that can accurately enhance the assessment of PCN risk. The main limitation is the limited number of studied patients, which depends on the monocentric nature of the study and the low prevalence of PCN cases that undergo the EUS-FNA procedure. Another notable limitation is the lack of prospective validation.

## 5. Conclusions

In conclusion, cystic fluid levels of glucose, CEA, LDL cholesterol, and total proteins, along with lymphocyte count, might contribute to the risk stratification of IPMNs and further clinical decision making, which can be further improved by metabolomic analyses. However, more prospective studies are necessary to validate these promising biomarkers in larger populations, facilitating earlier prognosis and diagnosis of PDAC patients.

## Figures and Tables

**Table 1:** Demographic, clinical, and laboratory characteristics of patients with high- and low-risk pancreatic cystic neoplasms (PCNs). Mean values ± Standard Deviations for continuous variables and absolute number (*n*) with percentages (%) for categorical variables are shown. The statistical analyses of data were made by Student’s *t* test and chi square for continuous and categorical variables, respectively.

Demographic, Clinical, and Laboratory Variables	High-Risk Group *n* = 14	Low-Risk Group *n* = 12	*p*-Value
**Age,** Years	74 ± 5	64 ± 11	**0.003**
**Gender**			
**Males**, *n* (%)	7 (50)	4 (33.3)	0.391
**Females**, *n* (%)	7 (50)	8 (66.6)	
**Family history of PDAC**, *n* (%)	1 (7.1)	1 (8.3)	0.720
**Previous cancers**, *n* (%) *	4 (28.6)	4 (33.3)	0.613
**Cyst size,** cm	3.45 ± 0.92	3.06 ± 0.91	0.300
**Cyst location**, *n* (%)			
**Head-Neck-Uncinate process**	4 (28.5)	0	
**Head**	6 (42.8)	7 (58.3)	
**Body-tail**	0	3 (25)	
**Uncinate process**	1 (7.1)	1 (8.3)	
**Head-Body-Tail**	3 (21.4)	1 (8.3)	
**Cyst focality**, *n* (%)			
**Single**	7 (50)	9 (75)	0.248
**Multiple**	7 (50)	3 (25)	
**String sign positive**, *n* (%)	8 (57.1)	3 (25)	0.240
**Diabetes**, *n* (%)			
**No diabetes**	8 (57.1)	6 (50)	0.788
**Diabetes**	3 (21.4)	2 (16.6)	
**Pre-diabetes**	3 (21.4)	4 (33.3)	
**S-CEA**, μg/L	3.80 ± 4.23	1.45 ± 0.73	0.060
**S-CA19-9**, kU/L	28.50 ± 24.57	8.82 ± 5.53	**0.027**
**P-Total Proteins**, g/L	70.07 ± 8.33	72.00 ± 6.67	0.526
**P-Albumin**, g/L	42.42 ± 5.54	44.33 ± 4.84	0.359
**P-Total Bilirubin**, mmol/L	9.31 ± 8.35	11.62 ± 8.93	0.502
**P-AST**, U/L	25.57 ± 8.88	24.33 ± 4.84	0.658
**P-ALT**, U/L	21.07 ± 8.49	19.83 ± 4.58	0.643
**P-ALP**, U/L	65.64 ± 16.28	71.58 ± 20.22	0.415
**P-gGT,** U/L	16.78 ± 10.07	29.08 ± 27.16	0.161
**P-Glucose**, mmol/L	5.88 ± 1.84	5.64 ± 1.00	0.686
**P-Cholesterol**, mmol/L	4.84 ± 0.98	5.27 ± 0.60	0.203
**P-LDL Cholesterol**, mmol/L	2.79 ± 0.76	2.94 ± 0.51	0.575
**P-HDL Cholesterol**, mmol/L	1.55 ± 0.35	1.69 ± 0.40	0.342
**P-Triglycerides**, mmol/L	1.09 ± 0.34	1.30 ± 0.43	0.189

* Previous cancers were breast, FAP, endometrial, and colorectal in low-risk and multiple myeloma and colorectal, adrenal myelolipoma, and thyroid in high-risk patients. S, serum; P, plasma.

**Table 2:** Circulating immune cell subsets in high- and low-risk IPMN patients.

Blood Values	High-Risk Group (N = 14)	Low-Risk Group (N = 12)	t	*p*-Value
**Hb (g/L)** Mean ± SD	131 ± 19	129 ± 16	0.260	0.797
**Platelets (10^9^/L)** Mean ± SD	216 ± 66	230 ± 87	0.478	0.637
**Neutrophils (10^9^/L)** Mean ± SD	3.48 ± 1.61	3.33 ± 0.86	0.264	0.794
**Lymphocytes (10^9^/L)** Mean ± SD	1.71 ± 0.53	1.90 ± 0.56	0.849	0.405
**Monocytes (10^9^/L)** Mean ± SD	0.42 ± 0.14	0.46 ± 0.17	0.705	0.489
**Mature T cells (%)** Mean ± SD	72 ± 8	70 ± 8	0.660	0.515
**T Helper Inducer (%)** Mean ± SD	46 ± 11	44 ± 9	0.536	0.597
**CTLs (%)** Mean ± SD	24 ± 8	24 ± 8	0.077	0.939
**B Cells (%)** Mean ± SD	8.5 ± 5.0	9.4 ± 4.6	0.483	0.633
**NK Cells (%)** Mean ± SD	18 ± 10	20 ± 10	0.273	0.787
**Non-MHC-Restricted Cytotoxic Cells (%)** Mean ± SD	9.6 ± 5.0	5.5 ± 2.8	2.509	**0.019**
**B-HSPCs (N/mL)** Mean ± SD	586.91 ± 315.29	784.59 ± 481.31	Chi square 0.074	0.786

**Table 3:** ROC curve of cystic fluid biochemical indices and of lymphocytes in distinguishing high- from low-risk PCNs.

Biomarkers	AUC	SE	95% CI		Best Cut-Off
**CEA (N = 24)**	0.8786	0.0723	0.73694	1.00000	52 μg/L
**Amylase (N = 24)**	0.5857	0.1399	0.31143	0.86000	558 U/L
**Glucose (N = 22)**	0.6250	0.1093	0.41074	0.83926	0.9 mmol/L
**Total cholesterol (N = 20)**	0.7240	0.1184	0.49199	0.95593	0.65 mmol/L
**HDL cholesterol (N = 19)**	0.6818	0.1186	0.44929	0.91435	0.09 mmol/L
**LDL cholesterol (N = 19)**	0.7955	0.1117	0.57644	1.00000	0.22 mmol/L
**Triglycerides (N = 20)**	0.7344	0.1133	0.51235	0.95640	0.12 mmol/L
**Total proteins (N = 20)**	0.8594	0.0945	0.67411	1.00000	12 g/L
**Lymphocytes (N = 20)**	0.8687	0.0879	0.69639	1.00000	30%

AUC, area under curve; SE, standard error; 95% CI, 95% confidence interval.

**Table 4:** Sensitivity, specificity, positive and negative predictive values of cystic fluid lymphocytes, and biochemical indices.

Biomarkers (Cut-Off)	Sensitivity 95% CI	Specificity 95% CI	PPV 95% CI	NPV 95% CI	Accuracy 95% CI
**Lymphocytes (≤30%)**	91% 58.7–99.8	78% 40.0–97.2	83% 59.2–94.5	88% 51.1–97.9	85% 62.1–96.8
**CEA (>192 μg/L)**	36% 12.8–64.9	100% 69.2–100	100% 47.8–100	53% 42.9–62.2	63% 40.6–81.2
**CEA (>52 μg/L)**	71% 41.9–91.6	100% 69.2–100	100% 69.2–100	71% 52.2–85.1	83% 62.6–95.3
**Glucose (≤0.9 mmol/L)**	100% 73.5–100	40% 12.2–73.8	67% 54.7–76.8	100% 39.8–100	73% 49.8–89.3
**LDL Cholesterol (≤0.2 mmol/L)**	100% 76.8–100	50% 15.7–84.3	73% 57.9–84.6	100% 39.8–100	79% 54.4–94.0
**Total Proteins (≤12 g/L)**	100% 73.5–100%	63% 24.5–91.5	80% 62.1–90.7	100% 47.8–100	85% 62.1–96.8

PPV, positive predictive value; NPV, negative predictive value; 95% CI, 95% confidence interval.

**Table 5:** Putatively annotated metabolites from cystic fluid samples most significantly different between low- (LR) and high-risk (HR) PCNs.

	Ion Description	Detected *m*/*z*	Predicted Formula	RT (min)	p ^a^	Ratio (HR/LR)
**DL-Carnitine**	[M + H]+	162.10994	C_7_H_15_O_3_	5.898	0.0154	0.209
AcCa (5:0)	[M + H]+	246.16996	C_12_H_23_NO_4_	2.036	0.0853	0.334
AcCa (6:0)	[M + H]+	260.1855	C_13_H_25_NO_4_	3.763	0.1042	0.308
AcCa (8:0)	[M + H]+	288.21675	C_15_H_29_NO_4_	7.992	0.0693	0.412
AcCa (10:0)	[M + H]+	316.24808	C_17_H_33_NO_4_	11.332	0.0114	0.146
AcCa (12:0)	[M + H]+	344.27928	C_19_H_37_NO_4_	13.610	0.0204	0.339
AcCa (12:1)	[M − H]−	342.2637	C_19_H_35_NO_4_	12.498	0.0083	0.149
AcCa (14:1)	[M + H]+	370.29493	C_21_H_39_NO_4_	14.394	0.0114	0.175
AcCa (14:2)	[M + H]+	368.27935	C_21_H_37_NO_4_	13.394	0.0019	0.100
AcCa (16:0)	[M + H]+	400.34171	C_23_H_45_NO_4_	16.545	0.0068	0.295
AcCa (18:1)	[M + H]+	426.35729	C_25_H_47_NO_4_	16.821	0.0268	0.669
L-Kynurenine	[M + H]−	209.09211	C_10_H_12_N_23_	1.258	0.0441	0.500
Methyl indole-3-acetate	[M + H]−	190.08613	C_11_H_11_NO_2_	6.864	0.0028	0.187
Indole 3 lactic acid	[M + H]+	206.08129	C_11_H_11_NO_3_	4.336	0.0083	0.123
Bilirubin	[M + H]−	585.27019	C_33_H_36_N_46_	10.379	0.0059	0.001
**Acetylcholine**	[M + H]+	146.11666	C_7_H_15_NO_2_	5.665	0.0441	0.256
**L-pyroglutamic acid**	[M + H]−	130.04979	C_5_H_7_NO_3_	6.372	0.0204	0.521
Pyruvic acid	[M − H]−	88.01611	C_3_H_4_O_3_	1.343	0.0268	0.570
**Pipecolic acid**	[M + H]−	130.08624	C_6_H_11_NO_2_	5.163	0.0346	0.324
**DL-glutamine**	[M + H]+	146.06818	C_5_H_10_N_2_O_3_	6.373	0.0204	0.511
**DL-leucineamide**	[M + H]+	131.11782	C_6_H_14_N_2_O	0.625	0.0154	0.293
**Anisole**	[M − H]−	108.05723	C_7_H_8_O	0.528	0.0083	0.141
DG (O-24:6)	[M + H]+	431.31521	C_27_H_42_O_4_	16.392	0.0028	0.064
DG (38:6)	[M + H]+	641.51054	C_40_H_70_N_2_P_2_	22.149	0.0204	0.306
9-HpODE	[M + H]+	311.22307	C_18_H_32_O_4_	16.442	0.0019	0.096
TG (31:3)	[M + H]+	563.42791	C_24_H_55_N_10_O_3_P	19.115	0.0083	0.127
TG (31:4)	[M + Na]+	561.41248	C_33_H_62_OS_2_	19.276	0.0041	0.111
TG (35:4)	[M + H]+	617.47437	C_33_H_64_N_2_O_8_	19.791	0.0028	0.109
**LPC (15:1)**	[M + H]+	518.28325	C_24_H_46_N_3_O_3_P_3_	3.281	0.0268	0.285
LPC (16:0)	[M + H]+	496.33924	C_23_H_45_NO_4_	19.848	0.0346	0.269
**LPC (17:1)**	[M − H]+	524.33196	C_21_H_46_N_7_O_6_P	3.131	0.0103	0.077

Putatively annotated (Level 2) metabolites. Bold font indicates metabolites from HILIC chromatography. ^a^ Univariate analysis (Mann–Whitney) between groups.

**Table 6:** ROC curve of cystic fluid metabolites in distinguishing high- from low-risk PCNs.

**Biomarkers**	**AUC**	**SE**	**95% CI**	
AcCa (14:2) (N = 18)	0.9221	0.0778	0.7695	1.000
AcCa (16:0) (N = 18)	0.8857	0.0834	0.7222	1.000
Bilirubin (N = 18)	0.8831	0.0870	0.7126	1.000
DG (O-24:6) (N = 18)	0.9091	0.0689	0.7741	1.000
TG (31:4) (N = 18)	0.8961	0.0827	0.7341	1.000
TG (35:4) (N = 18)	0.9091	0.0734	0.7652	1.000
9-HpODE (N = 18)	0.9221	0.0647	0.7953	1.000
Methyl Indole 3 acetate (N = 18)	0.9091	0.0712	0.7696	1.000
Propylparaben (N = 18)	0.9091	0.0712	0.7696	1.000
AcCa (14:2) and HpODE (N = 18)	0.9351	0.0668	0.8040	1.000

AUC, area under curve; SE, standard error; 95% CI, 95% confidence interval.

## Data Availability

Data available upon request.

## Supplementary Materials

The following supporting information can be downloaded at: https://www.mdpi.com/article/10.3390/cancers17040643/s1, Figure S1: Volcano plots for cystic fluid samples; Figure S2: Pathway analysis based on metabolites that displayed significant variations in the cystic fluid of low- and high-risk patients. (a) Metabolite set enrichment analysis using RaMP-DB (integrating KEGG via HMDB, Reactome, WikiPathways). (b) Metabolomic pathway analysis using the KEGG database; Table S1: Summary of clinical, laboratory, imaging, and follow-up characteristics of IPMN patients stratified by risk category; Table S2: Correlations between cystic fluid biochemical parameters; Table S3: FC_Discriminant metabolites; Table S4: Pearson’s correlation analysis of biochemical data and metabolomic results. Pearson’s correlation coefficients and *p* values are given; Table S5: ROC curve of cystic fluid biochemical index and metabolites in distinguishing high- from low-risk PCNs.

## Abbreviations

The following abbreviations are used in this manuscript:

AcCa	Acylcarnitine
ALT	Alanine aminotransferase
ALP	Alkaline phosphatase
AST	Aspartate aminotransferase
CA19-9	Carbohydrate antigen 19-9
EUS-FNA	Endoscopic ultrasonography coupled with fine-needle aspiration
GGT	Gamma-glutamyl transferase
HDL	High-density lipoprotein
HCT	Hematocrit
Hb	Hemoglobin
HMDB	Human Metabolome Database
IPMN	Intraductal papillary mucinous neoplasm
KEGG	Kyoto Encyclopedia of Genes and Genomes
LDL	Low-density lipoprotein
LPC	Lysophosphatidylcholine
MCH	Mean corpuscular hemoglobin
MCHC	Mean corpuscular hemoglobin concentration
MCV	Mean corpuscular volume
MCN	Mucinous cystic neoplasm
NLR	Neutrophil/lymphocyte ratio
PCNs	Pancreatic cystic neoplasms
PLR	Platelet/lymphocyte ratio
QC	Quality control
ROC	Receiver operating characteristic
RBC	Red blood cell count
