# Supplementary material for: Cystic Fluid Total Proteins, Low-Density Lipoprotein Cholesterol, Lipid Metabolites, and Lymphocytes: Worrisome Biomarkers for Intraductal Papillary Mucinous Neoplasms
Source: Cancers (Basel). 2025 Feb 14;17(4):643. doi: 10.3390/cancers17040643 (PMC11853297; doi:10.3390/cancers17040643)
Supplement: Supplementary file 1 [file cancers-17-00643-s001.zip › Figure S1 Volcano plots for cystic fluid samples_MS.pdf]

Figure S1 Volcano plots for cystic fluid samples.

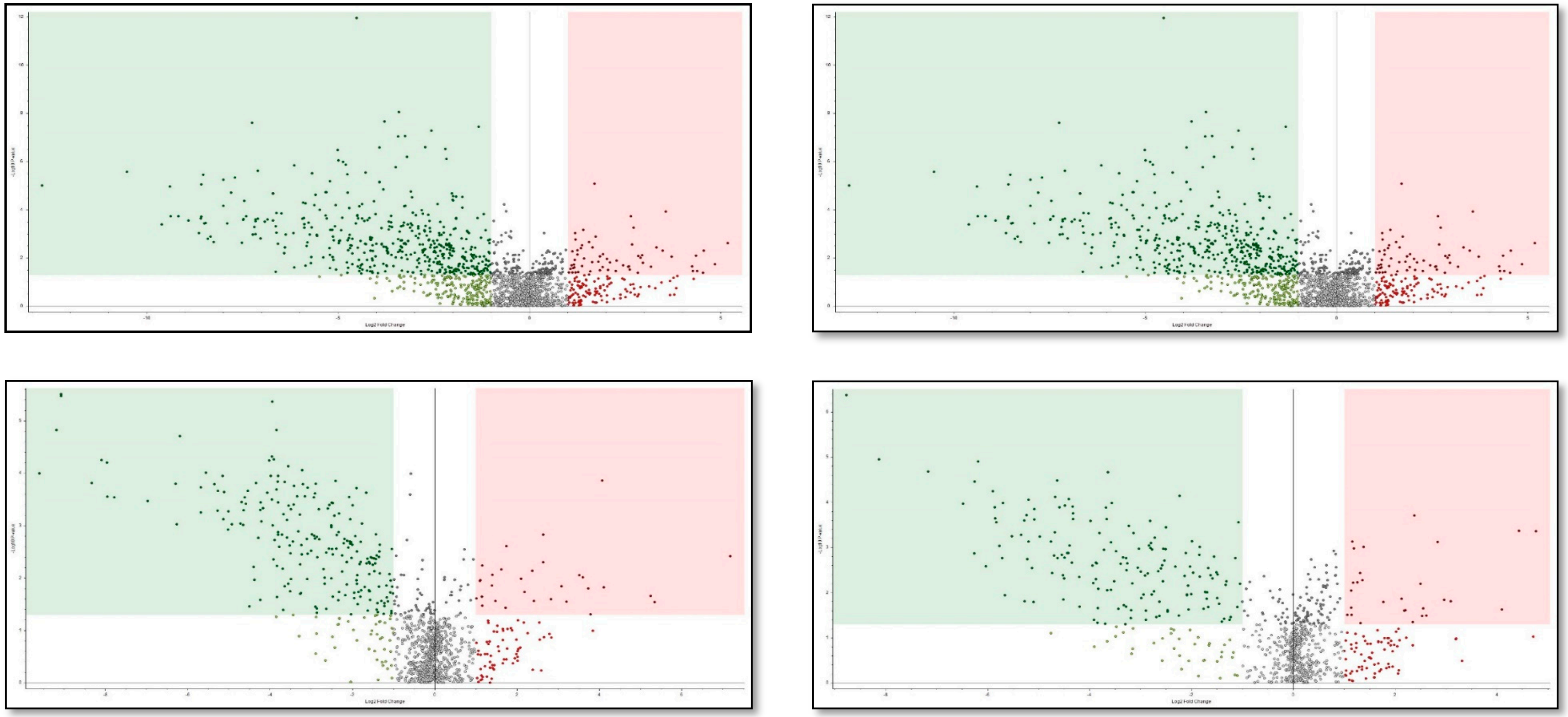

Volcano plot between high risk and low risk cystic fluid samples in positive (ESI+, panel on the left) and negative (ESI−, panel on the right) mode. HILIC compounds are in the upper part of the figure whereas, C18 compound are in the lower. Fold change (log2) on X-axis plotted against p-value (− log10) on Y-axis. The horizontal line marks the p = 0.05 and the vertical lines mark a fold change of ±1.0. Compared with the low risk samples, all plots in the upper green quadrant indicates a significantly downregulated metabolite in the high risk samples, whereas all plots in the right upper quadrant indicates the opposite.

Ratio= high risk/low risk samples
