# Supplementary material for: Cystic Fluid Total Proteins, Low-Density Lipoprotein Cholesterol, Lipid Metabolites, and Lymphocytes: Worrisome Biomarkers for Intraductal Papillary Mucinous Neoplasms
Source: Cancers (Basel). 2025 Feb 14;17(4):643. doi: 10.3390/cancers17040643 (PMC11853297; doi:10.3390/cancers17040643)

Supplemented Figure S2. Pathway analysis based on metabolites that displayed significant variation in the cystic fluid of low- and high-risk patients. (a) Metabolite set enrichment analysis using RaMP-DB (integrating KEGG via HMDB, Reactome, WikiPathways). (b) Metabolomic pathway analysis using the KEGG database

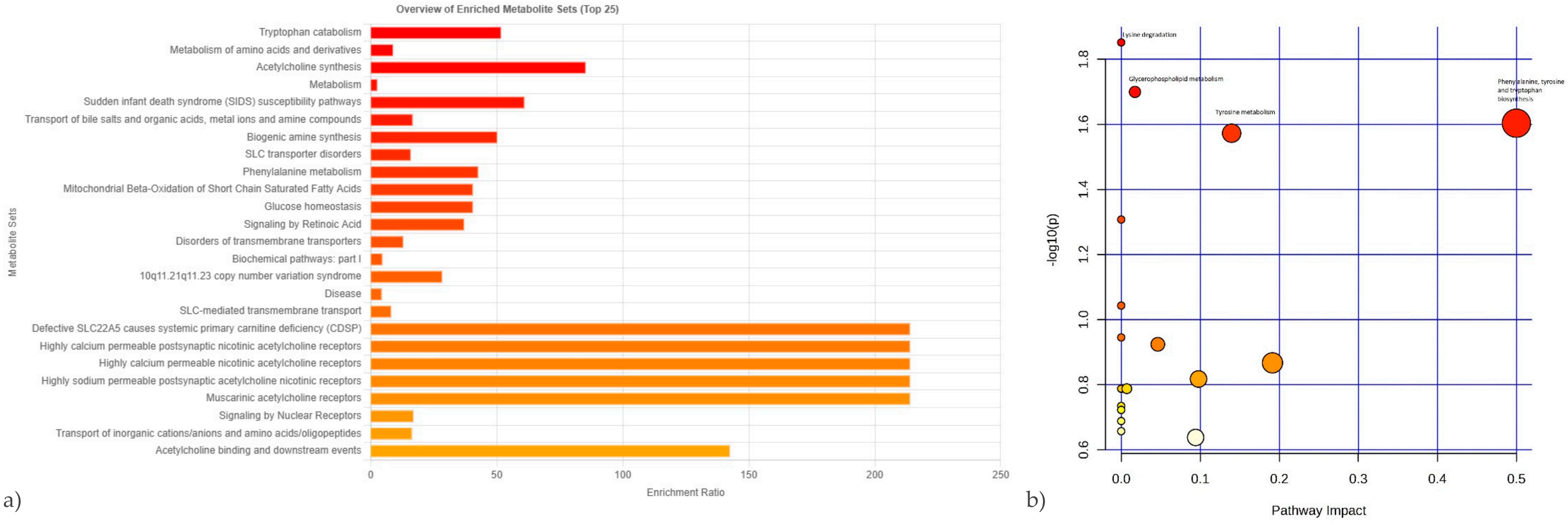

Supplement: Supplementary file 1 [file cancers-17-00643-s001.zip › Figure S2.pdf]
