# Supplementary material for: Cystic Fluid Total Proteins, Low-Density Lipoprotein Cholesterol, Lipid Metabolites, and Lymphocytes: Worrisome Biomarkers for Intraductal Papillary Mucinous Neoplasms
Source: Cancers (Basel). 2025 Feb 14;17(4):643. doi: 10.3390/cancers17040643 (PMC11853297; doi:10.3390/cancers17040643)
Supplement: Supplementary file 1 [file cancers-17-00643-s001.zip › Supplementary Table S1.pdf]

					Worrisome Clinical Features			Worrisome Imaging Features					
**Risk**	**Pts Nr**	**Gender**	**Age**	**Cytology**	**Acute Pancreatitis/Abrupt change in caliber of pancreatic duct with distal pancreatic atrophy/Lymphadenopathy**	**CA 19-9 > 37 KU/L**	**New onset or exacerbation of diabetes mellitus**	**Cyst ≥ 30mm**	**Enhancing mural nodules < 5 mm; thickened/enhancing cyst walls; main pancreatic duct (MPD) 5-10 mm**	**Cystic growth rate ≥ 2.5 mm/year**	**Follow-up before enrolment (years)**	**Positive Worrisome features (Nr)**	**Follow-up Surgery - Histology**
**High**	**1**	M	74	Negative	NO	Positive	NO	Positive	NO	Positive	3	3	
**Low**	**2**	F	76	Negative	NO	NO	NO	Positive	NO	NO	7	1	
**Low**	**3**	F	48	Negative	NO	NO	NO	NO	NO	N/A	0	0	
**Low**	**4**	F	70	Negative	NO	NO	NO	Positive	NO	NO	7	1	
**Low**	**5**	F	64	N/A	NO	NO	NO	NO	NO	N/A	0	0	
**Low**	**6**	F	57	N/A	NO	NO	NO	NO	NO	N/A	0	0	
**High**	**7**	F	84	Negative	NO	Positive	NO	Positive	NO	Positive	7	3	
**Low**	**8**	M	64	Negative	NO	NO	NO	NO	NO	N/A	0	0	
**Low**	**9**	F	57	Negative	NO	NO	NO	NO	NO	N/A	0	0	
**High**	**10**	M	73	Negative	NO	NO	Positive	Positive	NO	Positive	5	3	
**Low**	**11**	F	68	Negative	NO	NO	NO	NO	NO	NO	6	0	
**High**	**12**	F	82	Negative	NO	Positive	Positive	NO	MPD (9-10 mm)	NO	3	3	
**High**	**13**	F	76	N/A	NO	Positive	NO	Positive	NO	N/A	0	2	
**High**	**14**	F	75	Negative	NO	Positive	Positive	Positive	Hypoechoic wall thickening	N/A	0	4	

**High**	**15**	M	75	**Positive**	NO	NO	NO	NO	MPD (7 mm)	Positive	3	2	Yes - High grade dysplasia
**High**	**16**	F	70	Negative	NO	NO	NO	Positive	NO	Positive	1	2	
**High**	**17**	M	75	Negative	NO	NO	NO	Positive	NO	Positive	7	2	
**High**	**18**	M	67	**Positive**	NO	NO	NO	NO	NO	Positive	4	1	
**High**	**19**	M	68	Negative	NO	NO	NO	Positive	NO	Positive	1	2	
**Low**	**20**	M	56	Negative	NO	NO	NO	NO	NO	NO	4	0	
**High**	**21**	F	72	Negative	NO	NO	NO	Positive	NO	Positive	3	2	
**Low**	**22**	M	78	Negative	NO	NO	NO	NO	NO	NO	2	0	
**Low**	**23**	F	48	Negative	NO	NO	NO	Positive	NO	NO	6	1	
**High**	**24**	F	76	Negative	NO	NO	Positive	Positive	NO	N/A	0	2	
**Low**	**25**	M	80	N/A	NO	NO	NO	Positive	NO	N/A	0	1	
**High**	**26**	M	74	N/A	NO	Positive	NO	Positive	NO	Positive	6	3	Yes - Low grade dysplasia
