# Supplementary material for: Cystic Fluid Total Proteins, Low-Density Lipoprotein Cholesterol, Lipid Metabolites, and Lymphocytes: Worrisome Biomarkers for Intraductal Papillary Mucinous Neoplasms
Source: Cancers (Basel). 2025 Feb 14;17(4):643. doi: 10.3390/cancers17040643 (PMC11853297; doi:10.3390/cancers17040643)
Supplement: Supplementary file 1 [file cancers-17-00643-s001.zip › Supplementary Table S2.pdf]

Supplementary Table S2. Correlations between cystic fluid biochemical parameters.

Cystic Fluid Variables		Glucose (mmol/L)	Cholesterol (mmol/L)	HDL (mmol/L)	LDL (mmol/L)	Triglycerides (mmol/L)	Total Proteins (g/L)	Lymphocytes (%)	Amylase (U/L)
**Glucose**	r	1.00							
	p.value								
**Total Cholesterol**	r	0.667	1.00						
	p.value	0.003							
**HDL Cholesterol**	r	0.642	0.785	1.00					
	p.value	0.006	0.0001						
**LDL Cholesterol**	r	0.844	0.876	0.919	1.00				
	p.value	0.0001	0.0001	0.0001					
**Triglycerides**	r	0.774	0.930	0.731	0.854	1.00			
	p.value	0.0003	0.0001	0.0002	0.0001				
**Total Proteins**	r	0.656	0.749	0.451	0.679	0.831	1.00		
	p.value	0.0043	0.0001	0.046	0.0014	0.0001			
**Lymphocytes**	r	0.416	0.787	0.675	0.745	0.767	0.822	1.00	
	p.value	0.086	0.0002	0.003	0.0006	0.0003	0.0001		
**Amylase**	r	-0.454	-0.538	-0.469	-0.580	-0.517	-0.289	-0.519	1.00
	p.value	0.039	0.014	0.037	0.009	0.020	0.216	0.019	
