# Supplementary material for: Cystic Fluid Total Proteins, Low-Density Lipoprotein Cholesterol, Lipid Metabolites, and Lymphocytes: Worrisome Biomarkers for Intraductal Papillary Mucinous Neoplasms
Source: Cancers (Basel). 2025 Feb 14;17(4):643. doi: 10.3390/cancers17040643 (PMC11853297; doi:10.3390/cancers17040643)
Supplement: Supplementary file 1 [file cancers-17-00643-s001.zip › Supplementary Table S4.pdf]

Supplementary Table S4: Pearson's correlation analysis of biochemical data and metabolomic results. Pearson's correlation coefficients and p values are given.

	**Bilirubin**	**AcCa (12:1)**	**AcCa (14:2)**	**AcCa (16:0)**	**Methyl Indole 3 Acetate**	**TG (35:4)**	**TG (31:4)**	**DG (O-24:6)**	**Propyl paraben**	**9-HpODE**
**Triglycerides**	.6707<br>.0041	.6254<br>.0085	.6202<br>.0092	.8015<br>.0002	.6060<br>.0114	.6409<br>.0067	.6590<br>.0050	.6862<br>.0031	.6733<br>.0039	.7484<br>.0009
**CEA**	-.5287<br>.0241	-.6495<br>.0035	-.6660<br>.0026	-.2829<br>.2553	-.6288<br>.0052	-.6412<br>.0041	-.5803<br>.0116	-.5565<br>.0165	-.5679<br>.0140	-.7496<br>.0003
**Total Proteins**	.7822<br>.0004	.6280<br>.0084	.7182<br>.0018	.7358<br>.0012	.8060<br>.0002	.8586<br><.0001	.8361<br><.0001	.8486<br><.0001	.8147<br>.0002	.9000<br><.0001
**LDL Cholesterol**	.7849<br>.0006	.6951<br>.0039	.8054<br>.0004	.7865<br>.0006	.7896<br>.0005	.8070<br>.0003	.7896<br>.0005	.8196<br>.0002	.8259<br>.0002	.8133<br>.0003

TG, triglycerides; AcCa, acyl carnitines; DG, diglyceride
