# Supplementary material for: Cystic Fluid Total Proteins, Low-Density Lipoprotein Cholesterol, Lipid Metabolites, and Lymphocytes: Worrisome Biomarkers for Intraductal Papillary Mucinous Neoplasms
Source: Cancers (Basel). 2025 Feb 14;17(4):643. doi: 10.3390/cancers17040643 (PMC11853297; doi:10.3390/cancers17040643)
Supplement: Supplementary file 1 [file cancers-17-00643-s001.zip › Supplementary Table S5.pdf]

Supplementary Table S5. ROC curve of cystic fluid biochemical index and metabolites in distinguishing high from low-risk PCNs.

Biomarkers	AUC	SE	95% CI	
**AcCa (14:2) (N=18)**	0.9221	0.0778	0.7695	1.000
**AcCa (14:2) &TG**	0.9286	0.0729	0.7894	1.000
**AcCa (14:2) &Bilirubin**	0.9221	0.0778	0.7695	1.000
**AcCa (16:0) (N=18)**	0.8857	0.0834	0.7222	1.000
**AcCa (16:0) & TG**	0.9048	0.0774	0.7531	1.000
**AcCa (16:0) & Bilirubin**	0.8571	0.1206	0.6208	1.000
**Bilirubin (N=18)**	0.8831	0.0870	0.7126	1.000
**Bilirubin & TG**	0.9143	0.0756	0.7661	1.000
**DG (O-24:6) (N=18)**	0.9091	0.0689	0.7741	1.000
**DG (O-24:6) & TG**	0.9143	0.0676	0.7819	1.000
**DG (O-24:6) & Bilirubin**	0.8571	0.1213	0.6194	1.000
**TG (31:4) (N=18)**	0.8961	0.0827	0.7341	1.000
**TG (31:4) & TG**	0.9000	0.0800	0.7433	1.000
**TG (35:4) (N=18)**	0.9091	0.0734	0.7652	1.000
**TG (35:4) & TG**	0.9286	0.0648	0.8015	1.000
**9-HpODE (N=18)**	0.9221	0.0647	0.7953	1.000
**9-HpODE & TG**	0.9143	0.0705	0.7761	1.000
**9-HpODE & Bilirubin**	0.9351	0.0594	0.8186	1.000

**Methyl Indole 3 acetate (N=18)**	0.9091	0.0712	0.7696	1.000
**Methyl Indole 3 acetate & TG**	0.9429	0.0544	0.8363	1.000
**Methyl Indole 3 acetate & Bilirubin**	0.9481	0.0498	0.8505	1.000
**Propylparaben (N=18)**	0.9091	0.0712	0.7696	1.000
**Propylparaben & TG**	0.9286	0.0618	0.8075	1.000
**Propylparaben & Bilirubin**	0.9351	0.0594	0.8186	1.000
